# Is hydroxychloroquine beneficial for COVID-19 patients?

**DOI:** 10.1038/s41419-020-2721-8

**Published:** 2020-07-08

**Authors:** Xing Li, Ying Wang, Patrizia Agostinis, Arnold Rabson, Gerry Melino, Ernesto Carafoli, Yufang Shi, Erwei Sun

**Affiliations:** 1 Department of Rheumatology and Immunology, The Third Affiliated Hospital of Southern Medical University, No. 183, Zhongshan Avenue West, Tianhe District, Guangzhou, 510630 Guangdong, China; 2 Shanghai Institute of Nutrition and Health, Shanghai Institutes for Biological Sciences, Chinese Academy of Sciences, 320 Yueyang Road, 200031 Shanghai, China; 3 VIB-KU Leuven Center for Cancer Biology, KU Leuven, Leuven, Belgium; 4 Child Health Institute of New Jersey, Robert Wood Johnson Medical School, Rutgers University, New Brunswick, NJ USA; 5 TOR, University of Rome Tor Vergata, 00133 Rome, Italy; 6 Venetian Institute of Molecular Medicine, University of Padova, Rome, Italy; 7 The First Affiliated Hospital of Soochow University, State Key Laboratory of Radiation Medicine and Protection, Institutes for Translational Medicine, Soochow University Medical College, Suzhou, China; 8 Department of Rheumatology and Immunology, Shunde Hospital, Southern Medical University (the First People’s Hospital of Shunde, Foshan), 528000 Guangdong, China

**Keywords:** Drug safety, Innate immunity, Viral infection

## Abstract

The outbreak of coronavirus disease 2019 (COVID-19) caused by severe acute respiratory syndrome coronavirus 2 (SARS-CoV-2) was first reported in December 2019. As similar cases rapidly emerged around the world^1–3^, the World Health Organization (WHO) declared a public health emergency of international concern on January 30, 2020 and pronounced the rapidly spreading coronavirus outbreak as a pandemic on March 11, 2020^4^. The virus has reached almost all countries of the globe. As of June 3, 2020, the accumulated confirmed cases reached 6,479,405 with more than 383,013 deaths worldwide. The urgent and emergency care of COVID-19 patients calls for effective drugs, in addition to the beneficial effects of remdesivir^5^, to control the disease and halt the pandemic.

## US FDA approved hydroxychloroquine (HCQ) and chloroquine (CQ) for COVID-19 as an Emergency Use Authorization (EUA) with cautions issued soon after

On March 28, 2020, the U.S. Food and Drug Administration (FDA) issued an EUA to allow hydroxychloroquine sulfate and chloroquine phosphate donated to the Strategic National Stockpile (SNS) to be distributed and used for hospitalized COVID-19 patients. In fact, these two drugs have been used for decades for the therapy and control of malaria and autoimmune diseases.

In Peru, the bark extracts of cinchona tree was used to treat malaria and babesiosis started almost 400 years ago. About 200 years ago quinine was found to be the key anti-malaria compound in the bark. The analog of quinine, CQ was made in 1934 and formally introduced into clinical practice in the United States in 1947 for the prophylactic treatment of malaria. In addition, CQ was also used to treat rheumatoid arthritis, and lupus erythematosus. A safer derivative HCQ was made in 1955. In 2017, there were more than five million prescriptions of HCQ in the United States, indicating that in the absence of other drug interactions or special health conditions, HCQ should be a relatively safe drug.

Preliminary studies have suggested HCQ may have utility in fighting COVID-19^6,7^. Distinct possible effects may be related to its function in the treatment of COVID-19 patients: A. anti-virus, B. anti-inflammation, and C. anti-thrombotic. As until now there have been no data indicating HCQ has any immunity boosting effect, here we will mainly discuss the anti-virus and anti-inflammation effects. In in vitro assays, both CQ and HCQ have been shown to possess antiviral activity against various viruses, such as human immunodeficiency virus (HIV), hepatitis A virus, hepatitis C virus, influenza A and B viruses, influenza A H5N1 virus and others^8^. Recent studies reported that CQ and HCQ could also inhibit SARS-CoV-2 in vitro^9,10^, suggesting that they are potentially applicable to COVID-19 patients. However, there is to date no convincing report of the in vivo anti-viral effects of HCQ/CQ^11,12^. Several randomized controlled trials brought comforting news that CQ and HCQ showed potential effects in reducing respiratory symptoms and pulmonary inflammation as evaluated by computed tomography (CT) of COVID-19 patients^13^. Recently a French non-randomized open-label trial revealed that on day 6 the nasopharyngeal clearance of virus of the patients receiving HCQ/azithromycin, HCQ only, and the control group were 100%, 57.1%, and 12.5%, respectively^14^. In view of this report, the USA government argued that HCQ could be applied for treating COVID-19 and on March 28, 2020, the use of CQ and HCQ in COVID-19 patients was permitted by the US FDA^15^ and advocated by the Indian Council for Medical Research^16^, causing drug companies to ramp up CQ and HCQ production. This led to panic buying as people of attempted to acquire this purported “life saving drug”. Even some physicians stocked up CQ and HCQ for personal use in US and some European countries^17,18^. However, initially a lack of attention was paid to the risks of using CQ and HCQ^19^. Accordingly, on April 24, 2020, FDA cautions against the use of CQ/HCQ outside the hospital settings or a clinical trials. In fact, one person in the U.S. died and another became seriously ill after using verterinary formulation of CQ tablets intended for use in fish tanks in an effort to prevent COVID-19. Shortly after permitting the use of CQ and HCQ for treating COVID-19, the US FDA issued precautions on using these drugs. Hence, we believe that serious discussions of the potential mechanisms are urgently needed to guide the potential clinical application, evaluation of efficacy and prevention of adverse effects of these drugs.

### HCQ exerts strong immunomodulatory effects

**Fig. 1:**
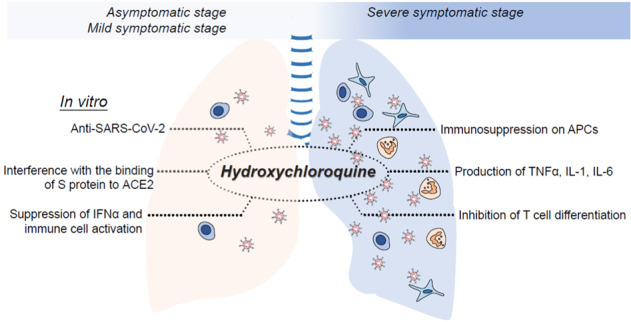
Hydroxychloroquine (HCQ) shows several potential effects against COVID-19 disease. While HCQ is likely to have the ability to control the CRS, suppress hyperactive immune responses and subsequently promote tissue repair, which leads to significantly improved severe symptoms in late-stage of COVID-19.

Despite widespread clinical use of CQ and HCQ in the treatment of inflammatory rheumatic diseases and virus infection, the underlying therapeutic effects and cellular mechanisms of these drugs remain largely unknown. Various modes of action have been proposed to explain the therapeutic and/or adverse effects of CQ and HCQ in COVID-19 patients, although most of the evidence is based on in vitro studies. CQ is a potent inhibitor of autophagy^20–23^ and cell death^24,25^, affecting distinct cell function^26,27^ and survival^28,29^, and its derivative HCQ^30^ has similar properties inhibiting autophagy. In vitro experiments in tissue culture have shown that CQ and HCQ can increase endosomal pH, prevent virus-cell fusion, and interfere with glycosylation of the ACE2 receptor and thus the binding of the SARS-CoV-2 S protein to ACE2^31^ (Fig. 1). On the other hand, we have proposed that the strong anti-inflammatory capacity of CQ and HCQ, which prevents autoimmune flare-ups and organ damages^32^, plays a more important role in controlling SARS-CoV-2 infection. The possible mechanisms of the anti-inflammatory effects of CQ and HCQ are mainly related to preventing antigen processing and interrupting molecular pathways involved in immune activation, subsequently resulting in the reduction of pro-inflammatory cytokine secretion^33,34^.

Various studies have shown that HCQ increases the intracellular pH and inhibits lysosomal activity in antigen-presenting cells (APCs), including plasmacytoid dendritic cells (pDCs)^35,36^ and B cells^37^, and also blocks major histocompatibility complex (MHC) class II-mediated antigen presentation to CD4^+^ T cells, and thus prevents the differentiation of these T cells (such as T follicular helper cells)^38^. This also leads to a reduction in the production of cytokines, such as tumor necrosis factor-alpha (TNF-α), interleukin 6 (IL-6), granulocyte macrophage colony-stimulating factor (GM-CSF), and IL-1β^38,39^. HCQ can effectively reduce symptoms in systemic lupus erythemat osus (SLE) patients by suppressing pDCs to secret pro-inflammatory cytokines induced by anti-dsDNA-associated immune complexes^40^. CQ also has been reported to block RNA-mediated TLR7 signal pathway activation^41,42^. In addition to TLRs pathway, CQ and HCQ also have effects on cyclic GMP-AMP (cGAMP) synthase (cGAS) activity by inhibiting its binding to cytosolic DNA, and thus down-regulating STING-dependent transcription of type I IFNs through IFN regulatory factor 3 (IRF3), ultimately leading to a reduction of type I IFN release^43–45^. Such mechanisms of CQ and HCQ in preventing antigen processing and suppressing inflammatory signaling pathways markedly reduced the production of pro-inflammatory cytokines, such as TNF-α, IL-6, and IFN-γ by mononuclear cells^39^, and IFN-α and CCL4 by pDCs treated with RNA-containing immune complexes^46^.

### Not all the patients are suited for taking HCQ against COVID-19

SARS-CoV-2 infection can be generally divided into three stages: asymptomatic, mild, and severe^3,47^. During the asymptomatic and early mild symptomatic stages, it is believed that if specific adaptive immune responses are developed, virus can be eliminated and disease will not progress to the severe stage to develop acute respiratory distress syndrome (ARDS)^47^. Therefore, boosting immune responses in patients in the early-stage or mild stage of infection is certainly the key to prevent progression to severe disease. Although CQ and HCQ have been reported to inhibit many viruses in vitro through either preventing virus–cell fusion or interfering with virus replication, the anti-viral activity of these agents has not been proven in any virus in any model in vivo. Therefore, the use of CQ or HCQ at early stages of disease may be related to eventual alternative effects; still, it may impinge on the production of type I IFN^43–45^ and the activation of immune cells^39,46^, which in turn could influence the development of the specific antiviral immunity.

Recent literature has pointed to the possibility that the number of asymptomatic infected proportion of a population may be larger than expected. Those asymptomatic individuals release substantial amount of SARS-CoV-2 virus and spread widely. The mechanism underlining the asymptomatic infection is unknown, however, if CQ or HCQ is used to prevent COVID-19, there could be undesirable consequences. Since CQ and HCQ could suppress the innate as well as adaptive immunity, application of these drugs when immunity is required may lead to the appearance of symptoms and spreading the virus to a larger population. The use of CQ or HCQ as prophylaxis for COVID-19 and the anticorrelation of CQ/HCQ usage in selected countries and COVID-19 morbidity are under study^48^.

We have proposed that at the severe stage of SARS-CoV-2 infection, inflammation is critical and leads to tissue damage, especially in the lungs^47^. At this stage, suppressing inflammation is likely to have therapeutic benefits. We propose that the anti-inflammatory and immunomodulatory effects of CQ and HCQ are the mechanisms of therapeutic effects that may be seen in COVID-19 patients at the severe stage. At this stage, through unknown mechanisms, large amount of cytokines are released and the patients develop cytokine release syndrome (CRS), or cytokine storm, an uncontrolled recruitment of immune cells and production of a unique combination of cytokines often in absence of T cells. These cytokines cause special type of ARDS within a very short period of time, requiring intubation and mechanical respiratory support^47^. This leads to severe damage to tissues of lungs, kidneys, and heart, and eventually results in a multiple organ dysfunction^49^. At this stage, CQ and HCQ treatment may be beneficial to reduce massive cytokine release by various immune cells through interfering with antigen processing and suppressing TLRs and cGAS-STING signaling. Such mechanisms provide support to the hypothesis that HCQ is likely to have the ability to control the CRS, by suppressing hyperactive immune responses and subsequently promoting tissue repair in COVID-19 patients (Fig. 1). Therefore, owing to the absence of solid evidence at this juncture, large scale, randomized controlled trials are necessary to assess the preventive and therapeutic effects of CQ and HCQ on asymptomatic, mild, and severe patients with COVID-19 to validate this hypothesis.

### Could HCQ and CQ have protective vascular effects in COVID-19 patients?

**Fig. 2:**
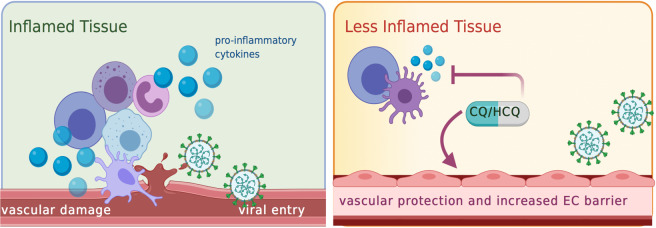
Speculative dual role exerted by HCQ/CQ on calming tissue inflammation and protecting the endothelium against SARS-CoV-2 mediated injury.

Vascular complications, including endothelium damage and vasculitis-like manifestations, are common traits in severe COVID-19 patients. In some patients vessel hyperplasia, vessel wall thickening, lumen stenosis accompanied by focal hemorrhage and thrombosis have been detected^3^. Conditions of severe vessel failure aggravate organ ischemia, tissue edema, and overall inflammation. This leads to the suggestion that SARS-CoV-2 may have a direct effect on endothelial cells (ECs), which also express ACE2 receptors. Such hypothesis is supported by findings showing that SARS-CoV-2 can indeed infect human blood vessel organoids^50^ and by post-mortem histological analysis of COVID-19 patient’s organs^51^ showing endothelitis and EC inflammatory cell death. These findings provide a strong rationale for the use of HCQ and CQ to alleviate these severe COVID-19 manifestations, since these drugs combine anti-inflammatory, anti-thrombosis^21,52^ and vascular protective effects^21^ (Fig. 2). We have previously shown that CQ has anti-angiogenic, tumor vessel normalizing properties in murine models of melanoma, without inducing EC death^21^. The EC effects induced by CQ included increased vessel barrier function, which alleviated tumor hypoxia. The vascular protective effects of HCQ and CQ, if validated, may be particularly relevant in patients with pre-existing diseases associated to vascular damage, like e.g. in diabetes, hypertension, and obesity.

### HCQ is more suitable than CQ in treating COVID-19

HCQ and CQ are extremely similar in their structure except for the addition of a hydroxyl group to the side chain and β-hydroxylation of the N-ethyl substituent. These modifications decrease HCQ toxicity while preserving its efficacy^53,54^. HCQ is administered as a sulfate, whereas CQ as a phosphate, and both of them are absorbed in the upper intestinal tract. The half-lives of CQ and HCQ are relatively long (960–1440 h) after absorption, and both drugs are shown to distribute to aqueous cellular and intercellular compartments, leading to long mean residence duration (~900 h for CQ and ~1300 h for HCQ)^55^. In general, both drugs are well tolerated. However, several common adverse effects have been reported in patients with long-term exposure to CQ and HCQ, such as gastrointestinal disorder, skin rash, retinopathy, blurred vision, cardiac toxicity, and others^56^ The most serious toxicity of HCQ and CQ is retinopathy, though it is rare, sight threatening may progress even to loss of vision and it is generally irreversible^57,58^. Clinical studies indicate that HCQ is associated with a lower risk of retinopathy than CQ, which may be due to the lower distribution volume as compared to CQ^55^. Another side effect of concern is cardiotoxicity caused by both drugs. Several researchers have reported cardiotoxic effects, such as myopathy, arrhythmia, and conduction disorder^59,60^. However, the exact evidence of cardiotoxicity caused by these drugs is still unknown. Moreover, keratopathy appears to occur more frequently in patients with CQ than with HCQ^61^. In addition, CQ exerts a number of severe side effects on fetal development, while HCQ can be safely used in patients with SLE during pregnancy and breastfeeding and provides protective effect for both mother and child^62^. The outbreak of SARS-CoV-2 has placed many pregnant women at high risk of infection (several infected cases have been reported). HCQ, rather than CQ, should be recommended as a more optimal choice for those patients, given its safety profile in pregnant women. Another important issue is whether CQ and HCQ are more toxic to COVID-19 patients is still a wide open question that need to be addressed.

### A cautionary note

However, recent observational studies in 1446 consecutive, non-randomized patients suggest that HCQ administration was not associated with either a greatly lowered or an increased risk of the composite end point of intubation or death^11^. Still, HCQ-treated patients were more severely ill at baseline than those who did not receive HCQ. More, the toxic side effects were minimal. In a retrospective multicenter cohort study in 25 different hospitals on 1438 patients with distinct medications and pre-existing conditions, there were no significant differences in mortality for patients receiving HCQ + azithromycin, HCQ alone, or azithromycin alone^12^. Another manuscript reported similar conclusion^63^, even though a serious “expression of concern” has been issued on this report^64^. Therefore, proper randomized, controlled trials of HCQ in patients with COVID-19 are needed. The multifaceted actions of HCQ on several vital processes, including autophagy and lysosomal function, which have been proposed to be key organ repair mechanisms essential to survive critical illness^65^, may ultimately oppose its potential benefits. Additionally, cardiotoxicity has been reported^66,67^. Also for this former paper, a serious “expression of concern” has been issued^68^. This still calls for caution, since besides elucidating ‘what and how’ further insights into ‘when’ HCQ should be administered need to be carefully examined. Therefore, further validation in randomized clinical trials is needed to establish both the efficacy of HCQ or CQ in reducing the vascular damage caused by SARS-CoV-2 and its therapeutic window in COVID-19 patients.

## Conclusion

Taken together, given the fast-increasing number of COVID-19 patients and the urgent need for effective and safe drugs in the clinic, CQ and HCQ have potential, but controversial, characteristics to combat pathological inflammation associated with COVID-19. The recommendation CQ and HCQ as a preventive medication for healthy and asymptomatic infected persons^48^, even for patients experiencing only mild symptoms in the early-stage of SARS-CoV-2 infection because of the immunosuppressive effects of the two drugs will diminish specific antiviral immunity, or as late stages therapeutic, still waits a proper double blind clinical trial. However, HCQ has been hypothesized to help controlling distinct effects of SARS-CoV-2 infection, as described above and compared to CQ, HCQ confers similar antiviral and anti-inflammatory effects while has fewer side effects, indicating HCQ is a more optimal selection for treating COVID-19. Importantly, when HCQ is used to treat COVID-19 patients, individual immune profiles should be thoroughly evaluated and considered. The above consideration offers a clear rational for a systematic evaluation of efficacy at the clinical level.
